# Future palliative competence needs – a qualitative study of physicians’ and registered nurses’ views

**DOI:** 10.1186/s12909-021-02949-5

**Published:** 2021-11-17

**Authors:** Arja Suikkala, Anniina Tohmola, Eeva K. Rahko, Minna Hökkä

**Affiliations:** 1 Diaconia University of Applied Sciences, Kyläsaarenkuja 2, FI- 00580 Helsinki, Finland; 2 Lapland University of Applied Sciences, Kemi, Finland; 3 Department of Clinical Oncology, Oulu University Hospital, Oulu, Finland; 4 Kajaani University Applied Sciences, Kajaani, Finland

**Keywords:** Palliative care, Palliative medicine, Palliative nursing, Professional competence, Qualitative research

## Abstract

**Background:**

Globally, the need for palliative care will increase as a result of the ageing of populations and the rising burden of cancer, non-communicable diseases as well as some communicable diseases. Physicians and registered nurses working in palliative care should have a sufficient level of education and competence in managing the changing needs and requirements of palliative care. There is, however, need for evidence-based palliative care training and education of physicians and registered nurses. The purpose of this study was to describe the views of physicians and registered nurses regarding future competence needs within palliative care.

**Methods:**

The study was conducted through use of a cross-sectional qualitative design. A total of 54 physicians and 110 registered nurses completed an open-ended questionnaire about the future competence needs of palliative care. The data were analyzed using inductive content analysis.

**Results:**

The results revealed four main competence needs within palliative care for the coming decade: palliative care competence at all levels within healthcare and social welfare services; individualized palliative care competence; person-centered encounters competence; and systematic competence development within palliative care.

**Conclusions:**

The results offer cues for education and professional development, which can be used to support physicians and registered nurses when future palliative care competences are included in educational programs. Seamless cooperation between palliative care services and educational institutions is recommended to ensure that undergraduate and postgraduate education is based on a continuous assessment of competence requirements within the field of palliative care. Therefore, online multi-professional simulations, for example, could be used to enhance future competencies within palliative care; undergraduate medical, nursing and allied healthcare students as well as postgraduate palliative care professionals and experts of experience could work together during simulations.

## Background

A central aim of palliative care is to provide high quality, seamless and affordable care to all patients and their significant others facing life-threatening diseases. Globally, approximately more than 56.8 million people require palliative care every year, but only 14% receive the proper care and support. Palliative care is increasingly provided in a wide range of social and healthcare settings for a growing number of palliative patients, ranging from mostly adults (67.1%) over 50 years old to children (7%) [1]. The COVID-19 pandemic has compounded the burden of suffering surrounding chronic diseases and thus highlighted the importance of palliative care competence among all healthcare professionals, especially when working among older people and people with preexisting risk factors to health [1, 2].

Palliative care plays a pivotal role in integrated and people-centered health services. This means that physicians and registered nurses (RNs) should understand themselves and act as members of a multi-disciplinary team when confronted with palliative care situations across healthcare and social welfare services. Worldwide, there is, however, neither any harmony of the training needed for palliative care nor the establishment of specialty or subspecialty palliative care medicine [1]. Furthermore, disparities in palliative education have been found across and between regions of Europe in undergraduate and postgraduate nursing programs [3].

Competence is defined as the holistic cluster of knowledge, skills, attitudes and values needed when delivering care effectively in a specific contextual situation [4–6] and is thus related to the provision of quality care [7] and consequently to job-satisfaction. Competence can be developed and improved via education and systematic professional development via formal and informal lifelong learning across the whole professional career [8–10]. Core competences for healthcare professionals, including physicians and RNs, have been determined in earlier literature with an aim to enhance the palliative care of patients and ensure multi-professional and inter-organizational collaborative work in all palliative settings. These competences range from all healthcare and social welfare professionals being able to work with a palliative care approach to those with discipline specific competences required in complex and demanding palliative care situations. The focus of competences includes early identification and assessment of the needs of the patient with life-threatening illness, delivering appropriate care based on the individual needs of the patient and ensuring the quality of life, i.e. by responding to the physical, psychological, social and spiritual needs of patients, communicating and collaborating with the patient, significant others and the range of professionals involved. Core competences are also seen as the ability to deal with loss, grief and bereavement by supporting and providing information, to enhance ethically high quality and appropriate palliative care to patients and their significant others [10–12].

Physicians and RNs should have a sufficient competence level of basic, advanced or specialized care, depending on whether they have adopted a palliative care approach, or work in generalist or specialist palliative care settings [8, 10, 13–15]. In general, physicians and RNs should have competence in patient-centered care including the ethical and legal aspects related to patient advocacy [16, 17], decision-making, and respect for the dignity and autonomy of palliative patients as persons with unique and wide-ranging needs, preferences, values and choices. Further, they need to have the competence to care and support an increasing number of patients with advancing chronic diseases in palliative care and their significant others [8, 9, 16, 17] in a wide array of disease groups, ethnic groups and patients who are marginalized or disadvantaged [15, 16, 18]. Collaboration with the patients, their significant others and multi-professional team as well as clinical and technical competence involve both generalist and specialist palliative care depending on which level the physicians and RNs work [16, 17]. In addition to the physicians’ abilities at the general level, those working at the specialized level of palliative care are supposed to have a strong competence in advanced symptom management, research and development competence, competence to offer consultative and educational support to other professionals and competence in providing a specialist level of psychosocial support [17]. Symptom management [10, 16, 19] providing holistic palliative care, and encountering and supporting the patient and their significant others [19] have been found to be the most important competences of RNs in both the basic and advanced levels of palliative care. In the advanced level, RNs’ competence in maintaining expertise and taking care of their own well-being at work has been underlined [10, 19].

Communication and relationships between everyone involved in palliative care, i.e. between healthcare professionals, students and patients and their significant others have been seen as being at the core of quality in palliative care [8, 9, 16, 19, 15, 20]. Competence regarding appropriate social interactions and multi-professional teamwork is emphasized as being important for both physicians and RNs [8–10, 16, 17, 19]. Effective communication, dialogue, active listening [16], sensitivity and empathy [19], in particular, have been highlighted among RNs [16], whereas competence in networking, consultation and the coordination of care pathways are seen as important for physicians [7].

Evidence-based palliative care and competence-based continuous personal and professional development are of great importance. Performing research and developing palliative care are seen as important competences of physicians [17] and specialized RNs [19]. Among RNs, attention needs to be paid to competences related to economic issues, quality control in palliative care [16] and participation in policy making [19]. For both physicians and RNs, it is important to continuously take care of and extend evidence-based professional competence and to also provide educational support to colleagues and other professionals [16, 17, 19, 21].

In the future, there will be an increasing need for palliative care professionals working in home- and community-based palliative care, which inflates the need for training in palliative care as well as multi-disciplinary collaboration competence with other professionals within and between palliative care levels [15, 16, 18]. There is, however, a dearth of evidence on future palliative competence needs regarding palliative care. The purpose of this study was to describe the views of physicians and RNs regarding future competence needs within palliative care.

The following research question was addressed:

What are the competence needs that will be required from physicians and RNs in palliative care during the next decade?

Ultimately, this study aims to contribute toward the continuous competence development of palliative care professionals and reinforce palliative care education in relation to future multi-disciplinary work.

## Methods

### Design, sample and data collection

This study was conducted in Finland using a cross-sectional qualitative design approach. The data were collected from a convenience sample of physicians and RNs by means of an electronic questionnaire sent via email between September and November 2018. The questionnaire had been developed by a multi-disciplinary team of palliative care experts including physicians (*n* = 2) with expertise in palliative medicine and a nurse researcher (*n* = 1) with expertise in developing undergraduate and special education in palliative care for nursing and medical students. The questionnaire was based on the EAPC recommendations of the palliative care content and future palliative care needs [14, 22]. The questions in the questionnaire were reviewed by an expert panel of representatives from palliative care nursing (*n* = 4) and palliative medicine (*n* = 3). This study focuses on one open-ended question in the questionnaire: What changes do you think will occur in physicians’ and RNs’ competence needs in palliative care and end-of-life care during the next decade?

The delivery of the invitation letter and the questionnaire was sent via email to 149 physicians with special competence in palliative medicine. Two reminders to complete the questionnaire were also sent. The mailings were arranged by a contact person of palliative medicine. The email addresses of the physicians were received from the register of The Finnish Medical Association. The response rates of physicians for the whole survey were 43.6% (*n* = 65) and 36% (*n* = 54) for the open-ended question concerning future palliative care competence needs.

As there exists no register of palliative care RNs in Finland, the data collection started by informing the stakeholders of healthcare organizations about the questionnaire via email and asking them to forward the message. In addition, the questionnaire was put on the EduPal (Developing Palliative Nursing and Medical Education through Multidisciplinary Cooperation and Working-life Collaboration) -project’s website and a link to the website was shared on the project’s Facebook page as well. A total of 132 RNs responded to the questionnaire and 83.3% (*n* = 110) responded to the open-ended question concerning future palliative care competence needs.

### Data analysis

Inductive content analysis was used to identify, code and categorize the data. The unit of analysis was a single word or meaning or a sentence that gave an answer to the research question. In the analysis, the researchers first read through the data to identify open codes which were further combined with other open codes referring to the similar content in order to form subcategories. Related subcategories were then combined to categories and main categories [23]. Two independent researchers conducted the analysis of each dataset (physicians’ and RNs’), reviewed each other’s analysis and, based on a joint assessment, placed the datasets together. The example presented in Table 1 illustrates the quotations, codes, subcategories, categories and main category.

**Table 1:** Example of the analysis process, considering person-centered encounters competence

Examples of quotations	Code	Sub-category	Category	Main category
“Palliative and end-of-life care is seen as important as any other treatment.” [MD 17] “The appreciation for palliative and end-of-life care will increase.” [RN 82] “Appreciation of encounters with dying patients will be emphasized.” [MD 21]	Palliative care as equally valuable as anything else. Emphasized appreciation of encounters with palliative patients.	Respect for palliative care patients.	Respecting encounters with patients and their significant others.	PERSON-CENTERED ENCOUNTERS COMPETENCE
“Patient encounters belong to all specialties and professional groups.” [MD 16]	Patients encounters belong to all specialties. Patients encounters belong to all professionals.	Significance in encountering palliative patients anywhere. Significance in encountering significant others.		
“The ability to face palliative and end-of life patients and their relatives will be increasingly emphasized”. [MD 21] “Warm presence.” [RN 19] “Interpersonal skills are important. We need time and expertise to meet each other.” [RN 11]	Ability to face palliative patients. Ability to face palliative patients’ significant others.			
“Everyone needs to have adequate communication skills.” [MD 43]	Adequate communication skills.			
“Specialist providers should still remember and be able to consider (patients’) quality of life and other issues in their work and patient education.” [MD 49]	Being able to consider the quality of life of patients. Being able to consider the quality of life in patient education.	Ensuring the quality of life of patients. Supporting psychosocial well-being.	Competence in delivering psychosocial support to patients and their significant others.	
“Increase in the proportion of lonely patients living without a social network.” [MD 51] “People are lonely.” [RN 64]	Increase in the proportion of lonely patients. Increase in the proportion of patients without any social network.			
“…more attention will be paid to mental and social factors.” [RN 46]	Attention to mental factors. Attention to social factors.			
“Nurses will need more psychosocial support training.” [RN 50]	Psychosocial support training. Internationalization. Different nationalities.	International competence. Multicultural competence. Religious literacy.	Competence in cultural and religious sensitivity.	
“… internationalization, different nationalities, religions.” [RN 97] “Finland is becoming more international, as well as patients.” [RN 84]				
“Different cultures need increasingly to be considered in care.” [MD 35]	Different cultures. Understanding of different cultures.			
“Understanding and knowledge of different cultures.” [RN 84] “Multiculturalism will increase.” [RN 71]	Knowledge of different cultures. Multiculturalism.			
“Multiculturalism and appreciation for different religions.” [RN 15]	Appreciation of different religions.			
“Different beliefs.” [RN 15] “Treating existential crisis.” [MD 39]	Appreciation of different beliefs. Treating existential crisis.			

*MD* abbreviation for Doctor of Medicine

*RN* abbreviation for Registered Nurse

### Ethical considerations

This study was held to the principles of ethical research [24, 25]. The permit to use email addresses from the special competency in palliative care register of physicians was obtained from The Finnish Medical Association. A covering letter attached to the front page of the questionnaire included information about the aim of the project and related questionnaire, voluntariness of the participation, what participation entails, anonymity and confidentiality. Both the physicians’ and RNs’ informed consent to participation was assumed by giving a response indicating that they had read the covering letter and agreed to participate by completing the online questionnaire.

## Results

### Participant demographics

Altogether, 54 physicians and 110 RNs completed an open-ended question about the future competence needs of palliative care. The participants represented diverse fields of general and specialized levels of palliative care [8, 10] covering public and private healthcare systems across Finland.

The mean age of the physicians was 51.3 years (range 34–69), most were women (92.6%) and all had qualified as a physician before the year 2010. Out of 54 physicians, 53 had special competence in palliative medicine and most also had specialist licenses in other fields of medicine. Out of 53 physicians, 51 worked within the field of palliative care, either in palliative care approach (18.4%), generalist palliative care (42.6%) or specialist palliative care (35.2%) (Table 2).

**Table 2:** Background data on physicians and nurses

Demographic variables	Physicians (*n* = 54)			Nurses (*n* = 110)		
	Mean (range)	n	%	Mean (range)	n	%
**Age (in years)**	51.3 (34–69)			44.9 (23–66)		
**Gender**						
Male		4	7.4		1	0.9
Female		50	92.6		105	95
Not defined /missing data					4	3.6
**Year of graduation**						
< 1979		4	7.4		2	1.8
1980–1989		12	22.2		15	13.6
1990–1999		26	48.1		25	22.7
2000–2009		11	20.4		34	30.9
2010–2019		0	0		34	30.9
Not defined /missing data		1	1.9			
**Special competences of physicians**						
Geriatrics		16	29.6			
General Practice		16	29.6			
Oncology and/or Radiology		15	27.8			
Anesthesiology, Intensive Care Medicine		5	9.3			
Respiratory Medicine and Allergology		2	3.7			
Pediatrics		2	3.7			
Other (Palliative Medicine, Obstetrics and Gynecology, Psychiatry, internal Medicine, Emergency Medicine)		5	9.3			
**Special competence in palliative medicine**						
Yes		53	98.1			
No		1	1.9			
**Nursing specialization**						
Medical-surgical nursing					30	27.2
Palliative and end-of-life care					13	11.8
Cancer nursing					11	10
Diaconal nursing					4	3.6
Midwifery					3	2.7
Pediatric nursing					2	1.8
Do not define/missing data					47	42.7
**Present work unit**						
at the basic level of service		10	18.4		49	44.5
at a specialized level of service		23	42.6		37	33.6
at an intensive tertiary level of service		19	35.2		17	15.5
Not defined /missing data		2	3.7		7	6.4

The mean age of the RNs was 44.9 years, most were women (95%) and over two-thirds (69.3%) had qualified as RNs before the year 2010. The RNs were working within the field of palliative care either in palliative care approach (44.5%), generalist palliative care (33.6%) or specialist palliative care (15.5%). More than half (57.1%) of the RNs had a specialized education (Table 2).

### Future competence needs within palliative care

Four main competence needs were formed regarding palliative care for the coming decade. Considerations will at first be given to palliative care competence at all levels within healthcare and social welfare services, then to individualized palliative care competence, person-centered encounters competence, and systematic competence development within palliative care (Table 3).

**Table 3:** Physicians’ and nurses’ views on future palliative care competence needs

Main category	Category
PALLIATIVE CARE COMPETENCE AT ALL LEVELS WITHIN HEALTHCARE AND SOCIAL WELFARE SERVICES	Palliative care competence across health settings. Competence in home-based palliative care and end-of-life care. Competence in working as a member of a multi-disciplinary team. Competence in the integration of palliative care consultation services with acute care units. ^a^ Competence in dedicated palliative care services. ^a^ Competence to provide needs in outpatient palliative care. ^b^
INDIVIDUALIZED PALLIATIVE CARE COMPETENCE	Competence in providing equal palliative care for all patient groups. Competence in dedicated palliative care in both malign and non-malign conditions and different patient groups. Competence in patient- and family-centered palliative care. Competence in symptom-based care. Competence in advanced care planning and hospice care. Competence in applying health technology. Competence in handling complex cases. ^a^ Competence in respecting human dignity and self-determination within palliative care. ^b^
PERSON-CENTERED ENCOUNTERS COMPETENCE	Respecting encounters with patients and their significant others. Competence in delivering psychosocial support of patients and their significant others. Competence in cultural and religious sensitivity.
SYSTEMATIC COMPETENCE DEVELOPMENT IN PALLIATIVE CARE	Competencies based on changing palliative care needs and requirements. Continuing competence assurance. Enhancing competence in palliative care through further and continuing education.

^a^Mentioned only in physicians’ data

^b^Mentioned only in nurses’ data

### Palliative care competence at all levels within healthcare and social welfare services

Physicians and RNs shared similar views on palliative care competence at all levels within healthcare and social welfare services with only a few exceptions. Palliative care competence at all levels within healthcare and social welfare services describes increased competence needs for palliative care across health settings from the basic level to the intensive tertiary level of services. The need for competence to provide quality in home-based palliative care and end-of-life care will increase in the future. Palliative care will be more common in patients’ homes and settings close to home, such as home nursing, long-term care, housing services, mobile and ambulatory care services and outpatient clinics.

*“Basic skills are needed in nursing homes”. (MD 50.)*

*“Palliative care at home will increase. This increases the need for special competence.” (RN 3.)*

Competence to work as a member of a multi-disciplinary team will be an important future need. Multi-professional collaboration, successful networking and consultations with other professionals to provide quality palliative care are increasingly seen as crucial in order to meet the physical, psychosocial and existential needs of palliative patients. Furthermore, physicians highlighted competence in dedicated palliative care services from the palliative care approach to the specialized level. The integration of palliative care consultation services with paramedics and acute care units was emphasized in order to ensure that the patients receive comprehensive care in their urgent healthcare needs. RNs, in turn, underlined the need for competence to provide care for the needs appearing in outpatient palliative care settings in the future.

*“Treatment of existential crisis.” (MD 39.)*

*“Nurses will need more psychosocial support training.” (RN 50)*

*“The involvement of emergency care providers in outpatient care will reduce unnecessary transfers of patients from their homes to the hospital.” (RN 23.)*

### Individualized palliative care competence

Individualized palliative care competence was characterized as providing equal palliative care for all patient groups regardless of the time of day, home setting or geographical location. Competence in dedicated palliative care in both malign and non-malign conditions and different patient groups such as children, cancer patients, chronically ill patients or patients with a severe impairment of well-being, multimorbid patients or intoxicant abuse patients were seen to be increasingly needed in the future. In addition, more attention needs to be paid to a growing elderly population and the required competencies that are needed when caring for their palliative care needs.

*“Patients with heart failure, chronic lung disease, and the elderly with multimorbidity are important groups of patients who also need quality palliative and hospice care.” (DR. 50).*

*“Equitable by providing the quality of care regardless of geographical location where patients live.” (RN 72.)*

*“The skills of doctors and nurses on a basic level need to be improved so that citizens receive equal services regardless of where they live.” (MD 11.)*

Competence in patient- and family-centered palliative care is needed even more in the future, especially in responding to the individual needs of palliative care patients and their significant others. Increasing awareness of palliative care, patients’ rights as well as increased health literacy among patients and their significant others, demand special competences to coherently respond to the individual needs of patients in their palliative care pathways. Therefore, listening to the patients and engaging with them and their significant others were seen as being of great importance. Besides encountering death and dying, competence related to aspects and possible statutes related to euthanasia were also seen as future competences.

*“The patient’s right to equal care is emphasized.” (MD 52.)*

*“Citizens’ awareness of treatment options is increasing and their demands are growing.” (RN 16.)*

Individualized palliative care competence was described as competence in symptom-based care, not only the management of pain but also the side effects of therapies at all levels of palliative care. Novel pharmacologic and non-pharmacologic symptom management interventions and palliative sedation require continuous competence development. Furthermore, competence in advanced care planning and hospice care will include competence in the coordination of palliative care, e.g. right-time advance care planning and implementation. Problem-solving and decision-making were also highlighted as future competencies for RNs.

*“Emerging symptomatic treatment techniques, e.g. pain management.” (MD 37.)*

*“Emphasis on end-of-life planning, individuality, consideration of wishes.” (RN44.)*

More emphasis will be placed on using health information technology, mobile health, and smart healthcare technology designed for the home of palliative care patients. As such, the utilization of telemedicine and smartphone-based applications will be increasingly needed both in professional consultations and collaboration with patients.

*“The use of electronic communication; e-consultations, better ways of networking.” (MD 57.)*

*“Artificial intelligence will provide solutions to a patient’s situation.” (RN 38.)*

Physicians considered the increasing importance of competence when handling complex cases in the future. These concerned patients’ and significant other’s wishes for futile treatments as well as discussions about difficult topics, e.g. wishes to end life or other ethical issues. RNs, for their part, highlighted competence in respecting human dignity and self-determination in palliative care.

### Competence in person-centered encounters

Competence in person-centered encounters was described in terms of respecting encounters with patients and their significant others anywhere in social welfare and healthcare services. Delivering psychosocial support to patients and their significant others was defined as ensuring the quality of life of patients and paying increasing attention to their psychosocial well-being due to the increase of loneliness and lack of social patient networks in the future. In addition, there is a need for competence in cultural and religious sensitivity in terms of international and multicultural contexts and therefore an increased need for religious literacy competence in any palliative care setting.

*“The need to be able to deal with palliative and end-of-life care patients and patients’ relatives is emphasized.” (MD 21.)*

*“Multiculturalism and respect for different beliefs.” (RN 15.)*

*“The different cultures as well as needs and preferences of patients and their significant others to be taken even more into account.” (MD 35.)*

### Systematic competence development within palliative care

Both the physicians and RNs felt that systematic competence development within palliative care was a crucial factor. As such, the competence needs and requirements at all levels of palliative care will increase all the time. This requires competence in service design, decision-making and economics. Managing the changing needs and requirements of palliative care constantly determines the competence requirements and requires continuing competence assurance. This is due to evolving palliative medicine and care as well as recommendations and regulations guiding the needed expertise, which in turn raises the need for human resources and expertise within palliative care, especially at the basic level of palliative care but also within the private sector. At the same time, patients and their significant others will become ever more aware of care, can demand care, and want to hear about their care.

*“Competence needs increase as the population is ageing.” (MD 5.)*

*“Self-directed learning and professional development will become an obvious matter.” (RN 39.)*

*“… recommendations and legislation may dictate issues related to education and professional development.” (RN 26.)*

It is essential to adopt a comprehensive approach to competence development within palliative care through further and continuing education. Systematic further and continuing education that is satisfactory in quality and quantity will be needed at all levels of palliative care. There will be an increased need for specialized education in palliative care, but also training about the basic level of palliative care for all health professionals. RNs will need education on evidence-based palliative care. In medical training, there is a need to include clinical training at the specialized level of palliative care services. Furthermore, more attention needs to be paid to professionals’ well-being at work.

*“Every professional must master the basic principles of caring for a dying patient (Level A care).” (MD 61.)*

*“In particular, training should be targeted at those working at level A care.” (RN 7.)*

*“Hopefully, the training will be systematic for both new nurses and doctors, plus those already in working life.” (RN 66.)*

*“Palliative care professionals need to take care of their well-being at work.” (MD 43.)*

## Discussion

In this study, the following four competence needs of physicians and RNs regarding palliative care for the next decade were found: palliative care competence at all levels within healthcare and social welfare services; individualized palliative care competence; person-centered encounters competence; and systematic competence development in palliative care. Palliative competences for curriculum development and continued professional development described in earlier literature are still seen as relevant in the coming decade [8–10, 16, 17, 19, 26], albeit also some new competence demands emerged.

Besides physicians and RNs, all social welfare and healthcare professionals need to have sufficient palliative education and competence to provide a high quality of palliative care. Therefore, palliative care educational programs are needed for the basic, advanced or specialized palliative care training of the workforce in order to align with palliative care needs, enhance the quality of palliative care and to ensure appropriate access to palliative care for all [1, 8, 9, 16]. In addition, the provision of high-quality palliative care necessitates competence development among those who work in the field of education, development and research [1, 19].

Palliative care competence is increasingly needed in home-based and out-patient palliative care settings, thus the need for the clinical education and practice of healthcare professionals is expanded from hospital-based care to more include home- and community-based palliative care [27]. There is also a growing need for the education and support of carers with regard to providing palliative care for those patients who wish to remain at home [18]. Thus, competence in collaboration and shared decision-making with patients, their significant others, carers and the range of professionals involved in planning and providing tailored palliative care is of great importance [10, 16, 17, 28].

Establishing palliative care competence in social and healthcare systems at all levels emphasizes leadership skills that enable seamless multi-disciplinary teamwork and inter-organizational collaboration within and between different levels of palliative care to ensure high quality care. More attention needs to be paid to competency-based, multi-professional palliative care education in order to ensure the amount and quality of professionals in general, but also with specialist palliative care competence, since clear roles, and effective and seamless multi-professional collaboration help in managing the challenges within palliative care [28, 29]. Simulations in palliative care education have been found to be an effective way to enhance interprofessional collaboration and are recommended for learning symptom management skills [30]. Integration of 24/7 palliative care consultation services with acute care units will increase in the future. This raises the need for new multi-professional approaches for both education and practice that will secure smooth pathways for responding to the acute care needs of patients. Furthermore, the expertise of physicians, RNs and other healthcare professionals who work in acute care or specialized level care will be needed more in consultative and educational support for those working at the general level of palliative care [3, 10, 16, 17, 19].

Individualized palliative care competence with an emphasis on patient- and family-centered palliative care was found as a second future competence among physicians and RNs. Dedicated palliative care in both malign and non-malign conditions and different patient groups will set competence requirements for complex symptom management at the specialized level. At the same time, there is an increasing competence need for providing equal and tailored palliative care for different patient groups, especially, indigenous, marginalized or disadvantaged and non-cancer patients [18]. Advanced care planning and decision-making take place in order to emphasize patients’ preferences to be cared for either in a hospice, hospital or increasingly in their own homes [1, 10, 17]. At the same time, an individual care plan helps relatives to cope, paving the way to a better end of life [31]. Previous literature has indicated technical aspects as part of palliative nursing clinical competency [16]. According to physicians and RNs in this study, telemedicine, smartphone-based applications and related technologies will enable home-based care in the monitoring and care of patients in the future. In some European countries, digital technologies have already been used to provide guidelines on symptom management and facilitate virtual contacts between palliative patients and their families [2]. This necessitates that healthcare professionals should be able to apply, and also critically assess the significance and impact of the digital technologies in consultations and interactions between healthcare professionals and patients [32]. Despite the increasing use of telehealth in healthcare, including palliative patients and their significant others, there is, however, a dearth of evidence in telehealth education and training [33]. This raises the need for appropriate telehealth education and pedagogical approaches, such as multi-professional simulations, team-based games [34], multi-professional healthcare escape rooms [35] for current and future social welfare and healthcare professionals working within palliative care settings.

Competence in person-centered encounters emphasizes uniqueness of patients and mutuality in patient-provider relationships, thus placing the focus of attention on the person with sensitive needs instead of the tasks [36, 37]. Emotionally intelligent person-centered encounter competence is needed to handle difficult thoughts and feelings with palliative patients and their significant others [16, 17]. In healthcare education, attention needs to be paid to the knowledge, skills, attitudes and values needed in person-centered encounters and dialogical relationships concerning a wide range of physical, emotional, social, spiritual and existential needs [16, 17, 28, 38]. The relievement of any existential and spiritual distress of patients as well as the grief and bereavement of significant others when facing loss, challenges professionals to improve their linguistic, cultural and religious literacy competence in a way that is sensitive and respectful to the individual patients and their significant others [39]. This raises the need for including a wide range of cultural as well as spiritual and religious issues in existing degree programs at all stages, as well as in continuing education courses. Competence in adequately dealing with complex situations, collaborating with other professionals and using the expertise of professionals with multi-cultural backgrounds in these situations are all of great importance [16, 40]. Inter-Professional Training Wards have been shown to enhance competences related to interprofessional skills and person-centered approaches [41]. In the future, special attention needs to be paid to recognizing the voice of palliative patients and their significant others [18] in the development and assessment of person-centered palliative care competence.

The changing palliative care needs and requirements necessitates the systematic development of competence and assurance in the field of palliative care. Healthcare professionals are obliged by Finnish law to maintain and develop their competence required for professional activities [42]. Continuous learning can occur formally, non-formally or informally [43] and education should be provided on different levels. This challenges educational institutions to provide further and continuing education aimed toward updating and renewing the competence and expertise of professionals working within palliative care. As palliative care competence is crucial for physicians and RNs worldwide, comprehensive, competency-based education will be needed to prepare them for practice with palliative patients and their significant others [3, 28]. In Finland, for example, new national curriculum recommendations for the education of physicians [44] and RNs [45] will harmonize the content of palliative education, highlighting what education should include.

### Strengths and limitations

This study had limitations related to the sample of physicians and RNs. All the physicians involved, except for one, had special competence in palliative medicine granted by The Finnish Medical Association. By comparison, only some of the RNs had a formal education in palliative care since there was no systematic education available. Therefore, both professionals have responded to the questionnaire based on their self-assessed expertise within palliative care. There may be a limitation of access to informants, and data collection via email might not have reached all 149 invited to the study. The sample of RNs consisted only of those respondents who had visited the website and answered the questionnaire, so this cannot be considered to be a completely comprehensive sample of palliative care physicians and RNs. There were twice as many RNs as physicians as informants, so one might think that the relationship is disproportionate. However, in practice, the number of palliative care physicians can be in a ratio of 1: 4 to the number of RNs [46]. The feedback from the physicians and RNs involved could have been used to ensure the creditability of the study, but this was not achievable.

## Conclusions

There is already a current need to pay attention to future competencies within palliative care, so that the palliative care competence of medical and nursing staff is accurate within all levels of healthcare and social welfare services in the next decade. Systematic and continuous competence development in all stages of palliative care is of great importance to ensure the quality of palliative care. This was the first study of the future palliative competence needs of physicians and RNs. Some of the competence needs have already been identified and some were new. More research is needed about future palliative care competencies, particularly the competencies required to provide palliative care for children and patients belonging to ethnic, linguistic, cultural and religious minorities. Providing increasing numbers of palliative patients with equitable access to quality palliative care will determine the competence and specific training needs of all social and healthcare professionals. In order to achieve the required competencies at all levels of palliative care, it would be advisable to incorporate the already established future palliative care competencies to education today.

## Data Availability

The data that support the findings of this study are available from the EduPal Project Manager but restrictions apply to the availability of these data, which were used under license for the current study, and so are not publicly available. Data are however available from the EduPal Project Manager upon reasonable request.

## Abbreviations

EduPal-project: Developing Palliative Nursing and Medical Education through Multidisciplinary Cooperation and Working-life Collaboration
MD: Doctor of Medicine
RN: Registered Nurse
